# The Inhibitory Effects of Naringin in a Rat Model of Postoperative Intraperitoneal Adhesion Formation

**DOI:** 10.1155/2022/5331537

**Published:** 2022-01-11

**Authors:** Xiaoqiang Shi, Yunhua Wu, Enmeng Li, Li Zhang, Yanfei Ma, Guangbing Wei, Xuqi Li, Shufeng Wang

**Affiliations:** ^1^Department of General Surgery, The First Affiliated Hospital of Xian Jiaotong University, Xian 710061, Shaanxi, China; ^2^Department of General Surgery, Shaanxi Provincial People′s Hospital, Xi′an 710068, Shaanxi, China; ^3^Second Department of General Surgery, The Suide Campus, The First Hospital of Yulin, Yulin 718000, Shaanxi, China

## Abstract

**Background:**

Many attempts have been made to inhibit the formation of postoperative intraperitoneal adhesions, but the results have been discouraging. Therefore, the identification of effective preventative measures or treatments is of great importance. In this study, the substantial potential of naringin (NG) to reduce peritoneal adhesions was validated in a rat model.

**Materials and Methods:**

A rat peritoneal adhesion model was established by abrasion of the cecum and its opposite intraperitoneal region under aseptic surgical conditions. After the operation, three groups of NG-treated rats were given 2 mL of NG by gavage at different concentrations (40, 60, or 80 mg/kg/d). The sham, control, and hyaluronan (HA) groups were given equal volumes of normal saline daily. On the 8th day, all rats were sacrificed 30 min after the administration of an activated carbon solution (10 mL/kg) by oral gavage. Intraperitoneal adhesion formation was adequately evaluated by necropsy, hematoxylin and eosin (HE) staining, Sirius red staining, immunofluorescence staining, enzyme-linked immunosorbent assays, and reactive oxygen species (ROS) probes. The gastrointestinal dynamics of the rats were assessed on the basis of a small intestinal charcoal powder propulsion test and the detection of motilin and gastrin levels in serum.

**Results:**

Intraperitoneal adhesions were markedly reduced in the group of rats receiving high-dose NG. Compared with the control group, the high-dose NG group showed clear reductions in inflammatory reactions, oxidative stress, collagen deposition, and fibroblast formation in the adhesion tissue and enhanced gastrointestinal dynamics (*P* < 0.05).

**Conclusion:**

NG alleviated the severity of intraperitoneal adhesions in a rat model by reducing inflammation, oxidative stress, collagen deposition, and fibroblast formation, highlighting the potential of NG as a drug candidate to prevent postoperative peritoneal adhesion formation.

## 1. Introduction

Postoperative intraperitoneal adhesion is a commonplace with high incidence rate, which could lead to severe complications, such as abdominal pain, intestinal obstruction, and intestinal necrosis, and threaten the postoperative recovery and long-term health of patients [1, 2]. However, until now, effective preventative measures and treatments for intraperitoneal adhesions have not been well developed.

Adhesions are fibrous bands that formed between nonanatomical sites. Previous studies have identified that intraperitoneal adhesion formation contains several mutual interactive and overlapping processes such as coagulation and fibrinolysis, inflammation, cell proliferation, and fibrosis [3]. Multifarious cell types such as immunocytes (such as macrophages, monocytes, and mast cells), fibroblasts, and mesothelial cells are involved in the above process [4]. It is recently suggested that the severity of inflammation is closely and positively correlated with adhesion formation. Treatments of inflammatory mediators antagonists, for example, IL-6, IL-17, and IFN-*γ*, prevent postoperative abdominal adhesion formation [5]. Our previous study has demonstrated that alleviating oxidative stress (ROS) conduces to inhibition of postoperative adhesion formation. [6] At present, it is generally accepted that myofibroblasts proliferate and infiltrate into the stable fibrin matrix (fibrin clot), then ECM proteins such as collagen start to deposit, and stable adhesion is finally formed [7].

The formation of intraperitoneal adhesions is common and inescapable after abdominal surgery [8], and they are also difficult to avoid even after minimally invasive laparoscopic surgery. Liquid or solid barriers are commonly used in clinical practice to prevent adhesion, but both of these material-based methods have disadvantages. Liquid barriers could drift to the outside of the damaged area and cause incision infections, peritonitis, and other adverse events [9], whereas solid barriers are easily displaced. The wide application of these methods is limited by their disadvantages. Other agents did not show sufficient efficacy [10]. Therefore, the development of novel and effective antiadhesion barriers and agents to solve these problems is of great importance.

In recent years, researchers have focused on natural products for the prevention of intraperitoneal adhesions due to their high availability, low cost, and significant biological activity [11]. For example, the efficacy of using *Bletilla striata*, *resveratro*l, and *ligustrazine* has been verified to prevent abdominal adhesion in rat models. Although these studies have only been conducted in animal models, such studies have revealed that natural drugs can exert anti-inflammatory effects, relieve oxidative stress, and balance fiber deposition and degradation to varying degrees, indicating that the research and development of natural drugs may provide a new exploratory direction for the prevention of intraperitoneal adhesions.

Naringin (NG; 4′,5,7-trihydroxyflavone-7-rhamnoglucoside) is a natural flavonoid (a flavanone glycoside) that has been shown to have anti-inflammatory, antioxidant, antiulcer, antiosteoporosis, and anticancer effects [12–14]. It can also inhibit fibroblast proliferation and motility, block the cell cycle, and promote fibroblast apoptosis [15]. Moreover, previous studies have found that NG can promote intestinal peristalsis and the recovery of gastrointestinal motility dysfunction in rats after surgery [16]. However, there have been no reports on the application of NG in intraperitoneal adhesions. Therefore, a prospective randomized controlled study of NG treatment in a rat model of intraperitoneal adhesion was performed to explore whether NG plays a role in preventing postoperative intraperitoneal adhesions.

## 2. Materials and Methods

### 2.1. Reagents

NG was purchased from Beijing Solarbio Science & Technology Co., Ltd. (Beijing, China), and was dissolved in 1.0% sodium carboxymethyl cellulose (1.0% CMC-Na; Shanghai Macklin Biochemical Co., Ltd., Shanghai, China) at different concentrations. Hyaluronate gel was purchased from Changzhou Institute of Medica Co., Ltd. (Changzhou, China). A TGF-*β*1 enzyme-linked immunosorbent assay (ELISA) kit was obtained from Wuhan Huamei Bioengineering Co., Ltd. (Wuhan, China). The ELISA kit for interleukin-1*β* (IL-1*β*) was purchased from Hangzhou MultiSciences (Lianke) Biotech Co., Ltd. (Hangzhou, China). ELISA kits for gastrin and motilin were purchased from Wuhan Cloud Clone Corp. (Wuhan, China). The malondialdehyde ELISA kit was purchased from Shanghai Zhen Ke Biological Technology Co., Ltd. (Shanghai, China).

### 2.2. Animals

Forty-eight adult male rats were purchased from the Experimental Animal Center of Xi′an Jiaotong University. All animals weighed between 200 and 250 g and were housed in standard laboratory conditions at 22 ± 2°C and fed standard pellet feed and tap water for drinking. This experiment was approved by the Biomedical Ethics Committee, Department of Medicine, Xi′an Jiaotong University (no. 2021-1591).

### 2.3. Experimental Design and Implementation

The rats were randomly divided into 6 groups, and the hair on their abdomens was shaved off one day before surgery. A standard adhesion model was employed as described by a previous study [17]. Briefly, anesthesia was induced by intramuscular injection of 25 mg/kg body weight ketamine. Then, the rats were placed on a sterile sheet. After the abdominal skin was disinfected, a 3 cm anterior midline incision was made in the abdomen of each rat, the skin and abdominal wall muscles were carefully separated, and the abdominal cavity was open. Next, the pouch-like cecum was located in the right lower abdomen. The cecum wall and its corresponding parietal peritoneum were rubbed with sterile gauze until petechial hemorrhages were observed. The abraded region was approximately 2-3 cm^2^ in size, and it was exposed to air for approximately 5 minutes. Finally, the cecum was placed in the anatomical part, and two injured surfaces were laid opposite. The abdominal wall muscle and the abdominal skin were sutured. In the sham operation group, only skin and muscle incisions were performed followed by suturing. The rats in the HA group were daubed in the abdominal cavity with 2 mL of medical hyaluronate gel before closing the abdominal wall.

Twenty-four hours after the operation, the rats in the NG1, NG2, and NG3 groups were orally administered with NG (40, 60, or 80 mg/kg, respectively). The NG dose was selected on the basis of NG doses that showed anti-inflammatory and antioxidant properties in other studies [18, 19], and each group was treated with the same dose, in the same manner, and at the same time each day. The other three groups were orally administered with an equal volume of normal saline at the same time as the NG groups were treated each day. One week later, all rats were fasted for 14 h and then orally administered with NG or saline in the same manner described above and then orally administered with 2 mL of charcoal powder solution (containing 5% charcoal powder and a 0.5% CMC-Na suspension) one hour later. After 30 min, 2 mL of blood from the heart of each rat was extracted and collected into vacuum tubes and centrifuged (13000 × g) at 4°C for 15 min, and the supernatant was stored in a −80°C freezer before the tests.

### 2.4. Evaluation of the General Adhesion of the Specimens

All rats were sacrificed, and a U-incision was made to open the abdominal cavity. Adhesion formation in each group was scored according to two independent observers′ blind evaluations. Nair′s scoring system was applied to evaluate the degree of severity of adhesion (Table 1). The rats were killed by excessive carbon dioxide inhalation as described previously [17], and specimens were collected for the following tests. In rats with adhesive tissue formation, the most severe site of adhesion formation included the visceral peritoneum of the cecum and the parietal peritoneum of the abdominal wall, and the three tissues mentioned above were collected to form a sandwich-like three-layer structure. In rats without adhesive tissue formation, the normal bowel wall of the cecum and right-side abdominal wall (used as normal peritoneal tissue) were removed and used as specimens [6].

### 2.5. Hematoxylin-Eosin Staining (HE)

One part of the specimen was steeped in formalin for 24 h and then cut into 4 *μ*m thick sections, while the other specimen was preserved in a −80°C freezer for intracellular reactive oxygen species (ROS) detection. Eight sections were randomly chosen from each rat tissue sample for HE staining, and five visual fields from each section were randomly chosen to determine the inflammatory response scores under high magnification. The average score per animal was considered the definitive score. All scores were assessed by two independent pathologists. The inflammatory response scores are summarized in Table 2.

### 2.6. Sirius Red Picric Acid Staining

Sirius red picric acid staining was used to observe the thickness of collagen deposition. Eight paraffin sections of each tissue sample were used for staining with a Sirius red picric acid staining kit (Anhui Leagene Biotechnology Co., Ltd., Anhui, China), complying with the kit's instructions. Five microscopic fields of each section were randomly selected to determine the collagen thickness. Collagen thickness was measured using Image-Pro Plus 5.0 software (Leica Qwin. Plus, Leica Microsystem Imaging Solutions Ltd., Cambridge, UK). The collagen thickness of each adhesive tissue sample was calculated as the average collagen thickness of the examined sections.

### 2.7. Immunohistochemistry

Immunohistochemical staining was used to assess the degree of *α*-SMA expression in the adhesion tissue. At least four paraffin sections of each adhesive tissue sample were randomly selected for dehydration and dewaxing, and then these samples were placed in citric acid antigen repair buffer for antigen repair. After completion, the sections were washed in PBS (pH 7.4) solution three times. Endogenous peroxidase was blocked with 3% hydrogen peroxide solution, and then the sections were washed again and incubated overnight with an *α*-SMA antibody at 4°C. Subsequently, the sections were incubated with the biotinylated secondary antibody diluted in antibody diluent and with the streptavidin conjugate for 30 min each at room temperature. Following washing, DAB coloration, and hematoxylin redyeing, ammonia treatment to adjust the staining level and neutral gum sealing were carried out. Five visual fields were randomly selected to observe the brown staining that indicated *α*-SMA protein expression under a microscope, and the immunohistochemical scores of each adhesive tissue were calculated as the average of all examined sections of each tissue sample. The protein expression scoring rules were as follows: 0 indicates no expression, 1 indicates weak positive expression, 2 indicates positive expression, 3 indicates strong positive expression, and 4 indicates extremely abundant expression.

### 2.8. ELISA

On the 7th day after the operation, the contents of IL-1*β* and transforming growth factor-*β*1 (TGF-*β*1) were measured as inflammatory indicators in adhesive tissues. Levels of malondialdehyde (MDA), an oxidative stress indicator in adhesive tissues, were measured. The levels of gastrin and motilin in serum were determined to evaluate the gastrointestinal dynamics status. Detection of these five indexes was performed strictly following the manufacturer′s instructions for each of the commercial ELISA kits. The concentrations of the samples were calculated from a standard curve.

### 2.9. Measurements of Intracellular ROS Levels

The DCF-DA method was used to measure ROS content in the adhesion tissues. Frozen sections from the adherent tissues or normal peritoneum of each group were made, and 8 frozen sections of each tissue sample were randomly selected. The tissue range was marked after dehydration and stained with ROS staining solution, and then the nuclei were stained with DAPI staining solution. The sections were incubated for 10 min at room temperature in the dark after washing, drying, and quenching the film to resist fluorescence. These sections were observed under a fluorescence microscope to collect images. Five high-magnification visual fields were randomly selected from each slice, and the fluorescence intensity of each slice was analyzed with imaging software. The average fluorescence intensity of each tissue sample was determined to be the fluorescence intensity of the tissue.

### 2.10. Assessment of Gastrointestinal Dynamics

All groups were subjected to charcoal powder propulsion tests on the 7th postoperative day to detect the differences in gastrointestinal motility between each group of rats. The experimental method was as described in our previous study [20]. The formula for intestinal propulsion rate is as follows: intestinal propulsion rate (%) = length of carbon powder movement/total length of small intestine × 100%.

### 2.11. Statistical Methods

All quantitative data are expressed as the mean ± SEM. One-way ANOVA and LSD tests were used to evaluate the differences among the groups of normally distributed data. The Kruskal–Wallis test was used to analyze the data without a normal distribution. Fisher's exact test was used to process the counting data. *P* values <0.05 were considered significant. All data were analyzed using SPSS 19.0 software.

## 3. Results

### 3.1. NG Treatment Reduces Intraperitoneal Adhesions

**Figure 1:**
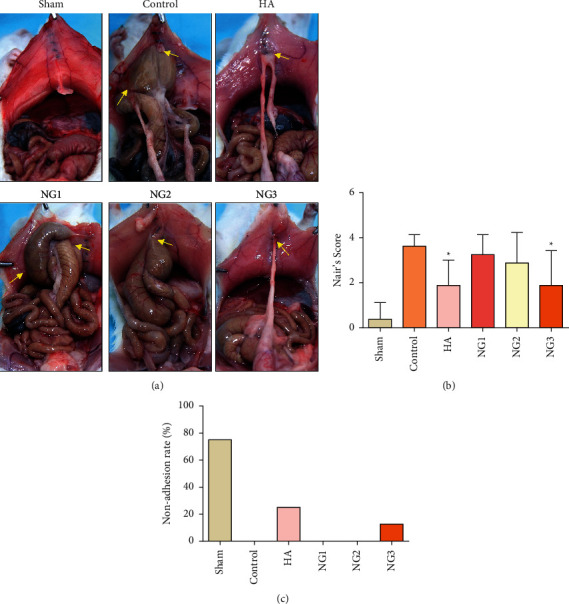
NG prevents intraperitoneal adhesion formation in rats on the 7th day after cecal abrasion (*n* = 8). (a) Intraperitoneal adhesions rarely formed in the sham operation group (sham). Dense and extensive adhesions were formed in all rats in the control group. Most of the rats in the sodium hyaluronate group (HA) formed only slight adhesions. All rats in the low-dose NG group (NG1) had severe adhesions. The degree of adhesion in the rats in the medium-dose NG group (NG2) was less than that in the NG1 group, and this trend was more pronounced in the high-dose NG group (NG3). One rat in the NG3 group had no adhesion formation. Yellow arrows in the images point to the adhesions. (b) The Nair scores of different groups. ^*∗*^*P* < 0.05 compared with the control group. (c) The no adhesion rate in each group.

All forty-eight adult male rats survived until the 7th day after the operation, and representative images of intraperitoneal adhesion formation in each group after the operation are shown in Figure 1. Adhesions were rarely observed in the sham group. However, extensive adhesions formed in the control group, mostly between intestinal tracts and the peritoneum or between intestinal tracts, and the adhesive bands were thicker compared with those in the HA group or NG groups. Low numbers of adhesions formed in the HA group, and these mostly formed between the omentum and peritoneum. The degree of adhesion in the NG treatment group decreased in a dose-dependent manner. Comparatively speaking, the adhesions in the NG3 and HA treatment groups were similar, and both groups had smaller and looser adhesion bands than the control group (Figure 1(a)), and the differences were statistically significant (Figure 1(b); *P* < 0.05). No adhesions were observed in 6 rats in the sham group, 2 rats in the HA group, and 1 rat in the NG3 group (Figure 1(c)).

### 3.2. NG Treatment Alleviates Inflammation in Adhesion Tissues

**Figure 2:**
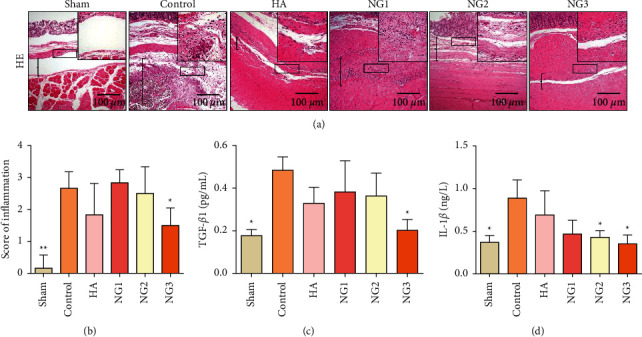
NG alleviates inflammation in rat adhesion tissues on the 8th day after surgery (*n* = 8). ^*∗*^*P* < 0.05 and ^*∗∗*^*P* < 0.01 compared with the control group. (a) HE staining of the different groups at 100× magnification; 200× magnification is shown in the upper right corner. The locations marked with brackets in the image are adhesion tissue. (b) The inflammatory score of each group was determined by HE staining. (c) TGF-*β*1 levels in the adhesive tissue were measured by ELISA. (d) IL-1*β* levels in the adhesive tissue were measured by ELISA.

To confirm the effect of NG on the inflammatory response during adhesion formation, HE staining of tissue specimens was performed. The numbers of inflammatory cells and the inflammatory score of the NG3 group were lower than those of the other groups except the sham group, and the differences compared with the control group were statistically significant (Figures 2(a) and 2(b); *P* < 0.05). Changes in inflammatory cytokine levels in adhesive tissues were further detected. Compared with the HA group, the low-dose NG group had no obvious advantage in the inhibition of TGF-*β*1 production but IL-1*β* production was inhibited to a greater extent. Compared with those in the control group, there was a significant decrease in TGF-*β*1 content in the NG3 group and IL-1*β* in the NG2 and NG3 groups (Figures 2(c) and 2(d); *P* < 0.05).

### 3.3. NG Treatment Decreases Collagen Deposition and Fibroblast Formation

**Figure 3:**
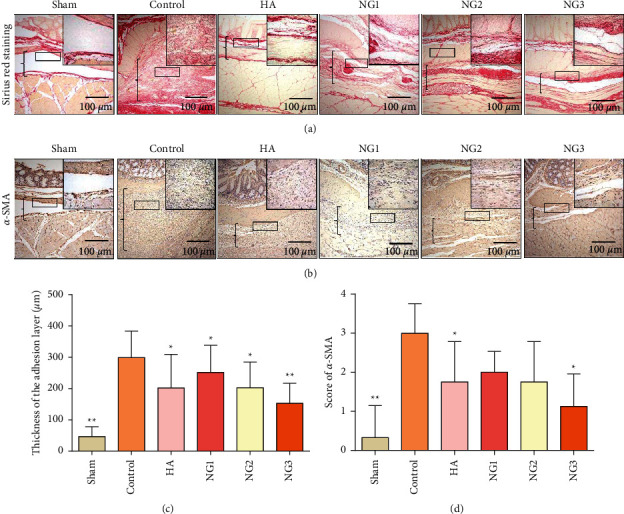
NG reduces collagen deposition during adhesion tissue formation in rats (*n* = 8). ^*∗*^*P* < 0.05 and ^*∗∗*^*P* < 0.01 compared with the control group. (a) The thickness of collagen deposition was measured by Sirius red picric acid staining (100×; insets, 200×). The locations marked with brackets in the image are the adhesion areas. (b) *α*-SMA expression in each group was measured by immunohistochemistry staining (100×; insets, 200×). The locations marked with brackets in the image are the adhesion areas. (c) Analysis of the adhesion thicknesses of each group by Sirius red picric acid staining. (d) The *α*-SMA expression scores in all groups.

Because the degree of collagen deposition and fibroblast formation partially reflects the severity of adhesion at the damaged site [5], we detected collagen thickness by Sirius red picric acid staining and the expression level of *α*-SMA (fibroblast-specific surface marker) in adherent tissues by immunohistochemical staining. Compared with the control group, the density of collagen and the thickness of the adhesion layer of the NG groups gradually decreased in a dose-dependent manner (Figures 3(a) and 3(c); *P* < 0.05). The immunohistochemical staining results showed that the *α*-SMA levels in the HA group and NG3 group were lower than those of the control group (Figures 3(b) and 3(d); *P* < 0.05).

### 3.4. NG Treatment Inhibits Oxidative Stress in Adhesion Tissues

**Figure 4:**
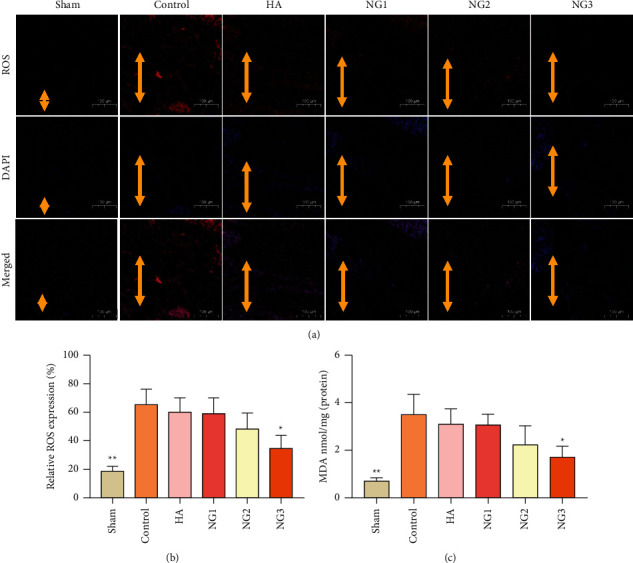
NG inhibits oxidative stress in the postoperative peritoneal adhesions in each group (*n* = 8). ^*∗*^*P* < 0.05 and ^*∗∗*^*P* < 0.01 compared with the control group. (a) Representative ROS levels of each group (100×). The lengths of the yellow double-sided arrows indicate the adhesion area. (b) The expression levels of ROS in the adhesion tissues. (c) MDA levels in the adhesion tissues were measured by ELISA.

Oxidative stress plays an adverse role in the repair of damaged tissues, and its severity can be reflected in the levels of ROS and MDA in adherent tissues [6]. Probes for ROS showed that the ROS level in the NG treatment groups decreased gradually in a dose-dependent manner (Figures 4(a) and 4(b); *P* < 0.05). The ELISA results showed that the MDA content in the adhesive tissues of the NG treatment groups also exhibited a decreasing trend compared with the control group, and the difference between the NG3 group and the control group was significant (Figure 4(c); *P* < 0.05).

### 3.5. NG Treatment Promotes Gastrointestinal Dynamics in Rats

**Figure 5:**
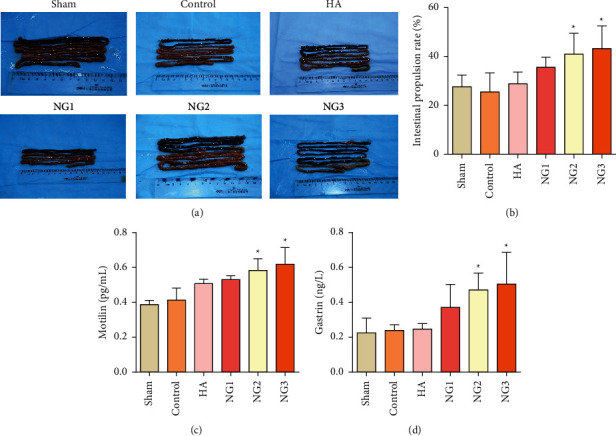
NG promotes gastrointestinal dynamics and gastrointestinal hormone secretion in rats. ^*∗*^*P* < 0.05 compared with the control group. (a) Representative images of the charcoal powder propulsion test show the state of intestinal motility in each group of rats. (b) The intestinal propulsion rates of each group determined by the charcoal powder propulsion test. (c) Motilin levels in the serum were measured by ELISA one week after the operation. (d) Gastrin levels in the serum were measured by ELISA one week after the operation.

To verify the effects of NG on gastrointestinal dynamics, we performed a charcoal powder propulsion test and simultaneously detected the secretion levels of gastrin and motilin in the blood. The results showed that the charcoal powder had moved further along the digestive tract in the NG groups than in the other groups (Figure 5(a)). The intestinal propulsion rates of the NG2 and NG3 groups were significantly higher than those of the control group (Figure 5(b); *P* < 0.05). Moreover, the secretion levels of gastrin and motilin in the NG2 and NG3 groups increased significantly compared with those of the control group (Figures 5(c) and 5(d); *P* < 0.05).

## 4. Discussion

Intraperitoneal adhesion is the most common complication after abdominal surgery, with an incidence of approximately 90–95% [21], which may cause distress or potentially mortality to the healthy life of patients during postoperative recovery. It is generally agreed that the mechanism of its occurrence is related to the inflammatory response, oxidative stress, an imbalance in the fibrinolytic system and fibroblast movement, and proliferation [22]. Therefore, any measure or drug that can reduce the severity of the inflammatory response and oxidative stress, accelerate the repair of the fibrinolytic system, or inhibit the proliferation of fibroblasts and their production of collagen in the damaged peritoneum will help to alleviate or avoid the formation of intraperitoneal adhesions. However, the current clinically used measures to prevent intraperitoneal adhesion are not satisfactory. In this study, we applied NG, a drug that is produced in large quantities in nature and that is safe to take orally for a long time, to a rat intraperitoneal adhesion model. The results showed that NG had a significant effect on reducing postoperative intraperitoneal adhesion formation.

The inflammatory response at the damaged site is one of the mechanisms that causes adhesion formation, which stimulates the exudation of a large number of inflammatory mediators from the peritoneum or intestinal wall, further stimulating the exudation and deposition of fibrin in the damaged area and leading to the formation of dense adhesions [4]. Second, the inflammatory response affects the normal repair of peritoneal mesothelial cells, promotes the transformation of mesothelial cells to mesenchymal cells, and contributes to adhesion formation [5]. To determine whether NG has anti-inflammatory effects on adhesion formation, we observed the degree of the inflammatory response in the adhesion tissue of different groups of rats by HE staining. Additionally, the levels of two inflammatory indexes, transforming growth factor-beta 1 (TGF-*β*1), which is considered the master molecule of adhesion formation and tissue fibrosis, and the proinflammatory cytokine IL-1*β*, were detected by ELISA. The results showed that the inflammatory response was mild in the adhesive tissue of the NG treatment groups, and the expression levels of TGF-*β*1 and IL-1*β* were lower in the NG groups than in the control group. The findings of this study suggest that NG plays an antiadhesion role by reducing the inflammatory response in intraperitoneal adhesion tissues.

Alpha-Smooth muscle actin (*α*-SMA) has been proven to be the hallmark of the transformation of fibroblasts into myofibroblasts in adhesion formation [23]; the latter have higher ability of remodeling extracellular matrix [24]. Some studies using fibroblast-populated collagen lattices demonstrated that the expression of *α*-SMA increases the contractile activity of fibroblasts and plays an irreplaceable role in the maturation of adhesion [25]. In a skin injury model, the skin wound contraction was deferred in mice with all-round loss of *α*-SMA and the scar was immature [26]. In addition, *α*-SMA expression regulates the proliferative activity of cardiac fibroblasts [27]. In embryonic stem cells, *α*-SMA expression may regulate cell fate [28].

It has been reported that, 3-5 days after surgery, fibroblasts invade the deposited fibrin clots to proliferate and secrete a large amount of collagen to form granulation tissue, which promotes the transition of loose adhesions firm and permanent dense adhesion [29]. NG was demonstrated to inhibit the movement and growth of fibroblasts during hypertrophic scar formation [19]. To determine whether NG inhibits the proliferation of fibroblasts, we detected the fibroblast marker *α*-SMA in adhesive tissues using immunohistochemistry, and the *α*-SMA score gradually decreased.

To identify the secretion of collagen at the damaged site, we measured the collagen content in different groups of adhesive bands by Sirius red picric acid staining. We can see that, with increasing NG dose, the collagen content in the adhesive bands gradually decreased, and the thickness of the adhesive bands gradually thinned.

The peritoneum is covered by mesothelial cells. Self-repair of mesothelial cells after injury is the key to reducing or avoiding the formation of intraperitoneal adhesion; otherwise, these mesothelial cells transform into mesenchymal cells (MMT) and promote adhesion formation, which has been implicated in numerous fibrotic disorders [30]. In addition to the inflammatory response, oxidative stress is another factor that prevents the self-repair of mesothelial cells. If the oxidative stress response of the damaged peritoneum can be inhibited or alleviated, adhesion will also be alleviated [6]. Based on this theory, in this experiment, we detected the ROS and MDA levels in adhesive tissue, which are two indicators of the degree of oxidative stress. It was confirmed that both ROS and MDA levels decreased in the adhesive tissues of the NG treatment groups, with more obvious results in the high-dose NG group. This study suggests that NG inhibited the degree of oxidative stress in adhesion tissues, and its mechanisms may be related to the ability of NG to ameliorate mitochondrial dysfunction [31, 32]; however, further experiments are needed for verification.

The contact site between the damaged peritoneum and the intestinal tube or the damaged intestinal tubes is the basis for intraperitoneal adhesion formation, and a barrier material can play a certain role in preventing adhesion based on this perception. We believe that if we can strengthen the peristalsis of the intestinal tube in the early stage of adhesion formation and promote the early recovery of gastrointestinal function, then reducing the contact time and area between the intestinal tube and the damaged peritoneum can also reduce the risk of intraperitoneal adhesion. In this study, we confirmed that the charcoal in the NG groups moved further than that in the other groups, reaching the distal end of the small intestine, and the secretion of motilin and gastrin increased significantly after high-dose NG treatment. This suggests that NG promotes gastrointestinal dynamics without causing complications due to intestinal obstruction. This pharmacological mechanism of action may be another explanation for NG-induced postoperative intestinal adhesion reduction.

Taken together, these results suggest that NG can be used as a drug to reduce the severity and incidence of adhesion, promote the early recovery of postoperative gastrointestinal function, and accelerate recovery after gastrointestinal surgery. However, some limitations still exist in our study. First, this study is based on animal models, and the effects of NG on the human body need to be further studied. Another limitation is the poor water solubility of NG, and most NG is degraded in gastric acid after oral administration, an issue that has not been addressed [33, 34], which may underestimate the efficacy of NG to prevent intraperitoneal adhesions. Hence, improving the controlled release and bioavailability of NG in the gastrointestinal tract has untapped potential.

## 5. Conclusion

NG can reduce the formation of intraperitoneal adhesions in rats by inhibiting the inflammatory response and oxidative stress, reducing fibroblast proliferation and collagen secretion, and promoting gastrointestinal peristalsis. NG may be a promising drug to prevent the formation of intraperitoneal adhesions.

## Figures and Tables

**Table 1:** Nair et al. scoring system.

Grade	Criteria
0	No adhesions
1	Adhesions between viscera or between visceral viscus and abdominal wall (one band)
2	Adhesions between viscera or between visceral viscus and abdominal wall (two bands)
3	Adhesions between viscera, between visceral viscus and abdominal wall (more than two bands), or multiple intestinal adhesions without adhesion to the abdominal wall
4	Direct adhesion of the viscera to the abdominal wall (the number and size of the bands are not important)

**Table 2:** Histopathological criteria for inflammatory score.

Score	Degree of inflammation
0	No inflammation
1	Giant cells, lymphocytes, and plasma cells
2	Giant cells, plasma cells, eosinophils, and neutrophils
3	Inflammatory cell infiltration and microabscess formation

## Data Availability

The data to support the findings of this study are available from the corresponding author upon request.
